# Customizing Yoga for Global Diversity: A Comparative Study of Eastern and Western Practices for Effective Integration Into Complementary Healthcare

**DOI:** 10.7759/cureus.71085

**Published:** 2024-10-08

**Authors:** Sanjay Gupta, Sony Kumari, Nick Vyas, Anjum Padyal

**Affiliations:** 1 Yoga Management, S-VYASA (Swami Vivekananda Yoga Anusandhana Samsthana) University, Bengaluru, IND; 2 Management, S-VYASA (Swami Vivekananda Yoga Anusandhana Samsthana) University, Bengaluru, IND; 3 Management, University of Southern California, Los Angeles, USA; 4 Kinesiology, California State University, Northridge, Northridge, USA

**Keywords:** cross-cultural comparison, cross-sectional studies, customizing yoga, east and west comparison, remote yoga, tele-yoga, tele-yoga therapy, yoga instructor, yoga intervention, yoga practice

## Abstract

Introduction: This cross-sectional study aimed to examine differences in yoga practices to enhance the validity and comparability of yoga research findings. It addresses the "black-box" approach to yoga interventions by highlighting various delivery components that can impact the validity of outcomes. By moving away from the generalization of yoga outcomes, this study provides deeper insights into yoga practices across culturally diverse populations. These insights are crucial for improving the reliability of outcomes and facilitating the integration of yoga into global complementary healthcare.

Methods: The study included 2,619 participants, with a balanced representation from India (1,296) and the United States (1,323). The participants were diverse, with 47.5% being yoga masters, gurus, therapists, and experts. The study utilized the 14 subscales of the Essential Properties of Yoga Questionnaire (EPYQ) to assess four factors of yoga practice: region (India/United States), sex (male/female), delivery mode (remote/in-person), and participation status (instructor/practitioner). The EPYQ demonstrated strong internal consistency (Cronbach’s alpha = 0.928).

Results: The total EPYQ score was higher in India than in the United States. Significant differences (p < 0.05) were observed for the 14 subscales across all four factors: region, sex, delivery mode, and participation status. Notable observations include that, by region, the United States sample scored higher than the Indian sample on the subscales of body awareness and acceptance/compassion (mean differences of -0.06 and -0.03, respectively), while the Indian sample scored higher on the spirituality and social aspects subscales (mean differences of 0.54 and 0.57, respectively). The remote delivery mode scored higher for the subscales of yoga philosophy and health benefits (mean differences of 0.17 and 0.13, respectively), and lower for the subscales of individual attention and active postures (mean differences of -0.17 and -0.04, respectively). By sex, the scores on the subscales of social aspects and yoga philosophy were higher for men than for women (mean differences of -0.26 and -0.24, respectively), whereas women scored higher for the subscales of body awareness and restorative postures (mean differences of 0.19 and 0.17, respectively). By participation status, practitioners showed higher scores for the subscales of physicality and active postures (mean differences of -0.05 and -0.02, respectively), whereas instructors scored higher on the subscales of yoga philosophy and meditation/mindfulness (mean differences of 0.37 and 0.32, respectively).

Conclusions: This study emphasizes the importance of recognizing variations in yoga practices and highlights the need for customization to enhance its integration into healthcare. To address the challenges posed by global diversity and practice heterogeneity, the study suggests moving from “black box” evaluations of yoga interventions toward data-driven analysis of macro- and micro-level factors. These insights can guide developers, healthcare providers, and researchers in creating culturally sensitive, user-friendly solutions and inform future cross-cultural research. Further related research will help create robust standards for yoga practices and delivery, applying the same rigor as that applied to conventional healthcare practices.

## Introduction

Yoga, a profound spiritual discipline and holistic health practice, has endured over the centuries, evolving to meet practitioners’ changing needs and transcending geographical and cultural boundaries [1]. Fundamentally, yoga integrates physical postures, breath control, meditation, and ethical principles to promote physical, mental, and spiritual well-being [2-4]. Integrated into modern healthcare, yoga enhances wellness [5], quality of life [6], and longevity [7], while helping to manage chronic conditions such as chronic pain [8], diabetes [9], heart disease [10], and cancer [11]. Additionally, the impact of yoga on mental health, including anxiety [12], depression [13], and the immune system [14], has been extensively studied. Thus, yoga has proven to be an effective complementary practice to conventional medical approaches, with the potential to contribute to universal healthcare goals.

Despite these benefits, significant challenges arise from global differences in yoga styles, demographics, delivery methods, and research methodologies. First, the diversity of yoga components, such as discipline practices (yama and niyama), physical postures (asana), breathing techniques (pranayama), and meditation styles, complicates the determination of cause-and-effect relationships and limits the generalizability of related research findings [15]. Second, yoga studies lack standardized procedures for selecting control and comparison groups, unlike drug intervention studies that often use placebo groups [16]. Third, yoga continues to evolve with modern adaptations and digital delivery technology, adding to its complexity [17,18]. Understanding these differences is crucial for establishing yoga as a reliable and effective health intervention, underscoring the need for a standardized framework for delivery and evaluation.

To enhance yoga’s efficacy, applications, and outcomes, this global cross-sectional study examined differences in yoga delivery from three perspectives. First, we evaluated yoga delivery using a large, diverse sample of practitioners from India and the United States to understand differences by region, sex, and role. Second, we compared the effectiveness of remote yoga versus conventional in-person delivery. The transition to remote yoga, accelerated by the COVID-19 pandemic [19-21], has led to skewed findings that may not apply to in-person settings [22]. Third, we compared the participation status of yoga users (instructors vs. practitioners). We employed robust and comprehensive instruments to assess the essential properties of yoga across this large sample.

By addressing the differences in yoga components and delivery modes, the objective of this study is to provide actionable insights to enhance the global comparability and validation of yoga practices, contributing to their integration into modern healthcare systems. Identifying component-level differences offers a roadmap for improving yoga experiences and outcomes. Additionally, understanding the impact of remote delivery is essential for establishing yoga as a reliable and effective health intervention.

This study bridges gaps in the literature by focusing on component-level differences in yoga practices between Eastern and Western populations. This analysis is essential for establishing a systematic and accurate diagnostic approach to yoga, ensuring that interventions, practice design, and delivery are precisely customized for target demographics to achieve optimal outcomes.

## Materials and methods

Survey design

This study used an online survey targeting yoga instructors and practitioners in India and the United States, conducted between April 2023 and March 2024. The survey design adhered to the Checklist for Reporting Results of Internet E-Surveys (CHERRIES) checklist and Strengthening the Reporting of Observational Studies in Epidemiology (STROBE) guidelines [23,24]. The questionnaire was administered via Google Forms, prioritizing user-friendliness with clear instructions and allowing participants to revise their responses. Electronic informed consent was obtained from all participants before the survey. Ethical approval was granted by the ethics committee of S-VYASA (RES/IEC-SVYASA/232/2022) on June 20, 2022. Participants were provided with comprehensive instructions to ensure accurate completion, focusing on their personal yoga experiences. Demographic information, yoga preferences, practice characteristics, and perceptions of remote versus in-person delivery modes were collected. Quality control measures included mandatory qualifying questions to prevent incomplete submissions and minimize random responses.

Participants and data collection

Stratified random sampling was employed to recruit participants from yoga institutes, studios, and organizations through direct engagement via social media, LinkedIn, email, search engines, and phone calls. Subgroups of providers and practitioners (beginners, intermediate, advanced) from each region were targeted. This approach aimed to reach a broad network of individuals with diverse demographics, yoga practices, and experiences with different yoga delivery modes. Inclusion criteria required participants to be 18 years or older, have at least one year of yoga practice, and the ability to complete the survey online. After removing outliers, the final sample included 2,619 individuals for analysis.

Measures

The Essential Properties of Yoga Questionnaire (EPYQ) was used to assess the scope and quality of yoga practice delivery [25]. The EPYQ is a robust tool for evaluating yoga instruction [26]. The questionnaire asked participants about their perceptions, delivery modes, and experiences regarding how comprehensively yoga was taught during sessions. This scale covers traditional yoga practices, including mind, body, and consciousness.

The EPYQ includes 14 subscales representing key components of yoga practice: acceptance/compassion, breathwork, physicality, active postures, restorative postures, body locks (bandhas), body awareness, mental/emotional awareness, health benefits, individual attention, social aspects, spirituality, meditation/mindfulness, and yoga philosophy. Participants rated their experiences on a 5-point Likert scale ranging from 1 (Not at all) to 5 (A very large amount), with higher scores indicating a stronger emphasis during yoga sessions [26]. The mean scores across these 14 subscales were calculated to provide an overview of the content and effectiveness of the delivered yoga sessions. The 14 subscales included 62 items assessing the content and coverage of yoga practices. The validity and reliability of the EPYQ were confirmed through confirmatory factor analysis and reliability testing with an initial subset of 100 responses.

Data analysis

Data analysis included demographic analysis, descriptive statistics, t-tests, chi-square tests, and multivariate analysis of variance (MANOVA). One-sample t-tests were used to determine the significance of scale and subscale items, focusing on identifying positive responses (scores above three). Chi-square tests were performed to assess internal validity. MANOVA was used to compare item, subscale, and overall scale scores between participants from India and the United States, as well as to examine differences by the four factors: region, sex, delivery mode, and participation status.

## Results

Yoga demographics

Among the 2,619 participants, there was a balanced distribution between the Indian (1,296) and U.S. (1,323) samples (Table 1). The EPYQ scale demonstrated strong internal consistency and content validity, with standardized correlation coefficients for the subscales ranging from 0.287 to 0.787, confirming its reliability (Table 2). Consistent with previous yoga studies, 65.5% of respondents were women. Over 79% of participants had at least a bachelor’s degree, and 31% had pursued formal graduate or higher education in yoga. The proportion of those seeking professional yoga education was higher in India (23%) compared to the United States (8%). Notably, 47.5% of participants held esteemed positions such as yoga masters, gurus, therapists, or experts, reflecting the growing recognition of yoga in global professional education. In terms of experience, 58.8% of participants had been practicing yoga for over five years, with 34.6% in the United States and 24.2% in India. Additionally, 36.2% of participants adhered to specific yoga lineages or traditions. The age distribution revealed that 82.9% of participants were between 18 and 55 years old, indicating that yoga is a popular lifestyle activity among this age group.

**Table 1:** Population Demographics

Factors		Region		Yoga Delivery Mode		
		India	North America	Remote	In-Person	Total
Delivery mode of participants	Online mode only	662 (25.3%)	513 (19.6%)	1175 (44.9%)	0 (0.0%)	1175 (44.9%)
	In-person mode only	634 (24.2%)	810 (30.9%)	0 (0.0%)	1444 (55.1%)	1444 (55.1%)
India or North America	India	1296 (49.5%)	0 (0.0%)	662 (25.3%)	634 (24.2%)	1296 (49.5%)
	North America	0 (0.0%)	1323 (50.5%)	513 (19.6%)	810 (30.9%)	1323 (50.5%)
Connection to Yoga	Student–Undergraduate	294 (11.2%)	116 (4.4%)	161 (6.1%)	249 (9.5%)	410 (15.7%)
	Yoga Scholar–MS, PhD	200 (7.6%)	55 (2.1%)	138 (5.3%)	117 (4.5%)	255 (9.7%)
	Regular Practitioner	329 (12.6%)	381 (14.5%)	369 (14.1%)	341 (13.0%)	710 (27.1%)
	Yoga Teacher, Master or Guru	314 (12.0%)	572 (21.8%)	348 (13.3%)	538 (20.5%)	886 (33.8%)
	Yoga Expert and Professors	159 (6.1%)	199 (7.6%)	159 (6.1%)	199 (7.6%)	358 (13.7%)
Experience of practicing yoga	Less than 2 years	257 (9.8%)	247 (9.4%)	242 (9.2%)	262 (10.0%)	504 (19.2%)
	2–5 years	405 (15.5%)	169 (6.5%)	308 (11.8%)	266 (10.2%)	574 (21.9%)
	5–10 years	237 (9.0%)	161 (6.1%)	192 (7.3%)	206 (7.9%)	398 (15.2%)
	Over 10 years	397 (15.2%)	746 (28.5%)	433 (16.5%)	710 (27.1%)	1143 (43.6%)
Follow yoga lineage (Guru)	Yes	512 (19.5%)	437 (16.7%)	434 (16.6%)	515 (19.7%)	949 (36.2%)
	No	571 (21.8%)	747 (28.5%)	584 (22.3%)	734 (28.0%)	1318 (50.3%)
	Maybe	213 (8.1%)	139 (5.3%)	157 (6.0%)	195 (7.4%)	352 (13.4%)
Age group	10–25 yr.	412 (15.7%)	145 (5.5%)	237 (9.0%)	320 (12.2%)	557 (21.3%)
	26–35 yr.	298 (11.4%)	245 (9.4%)	266 (10.2%)	277 (10.6%)	543 (20.7%)
	36–45 yr.	266 (10.2%)	263 (10.0%)	287 (11.0%)	242 (9.2%)	529 (20.2%)
	46–55 yr.	219 (8.4%)	324 (12.4%)	232 (8.9%)	311 (11.9%)	543 (20.7%)
	56–65 yr.	65 (2.5%)	226 (8.6%)	91 (3.5%)	200 (7.6%)	291 (11.1%)
	66–75 yr.	25 (1.0%)	94 (3.6%)	46 (1.8%)	73 (2.8%)	119 (4.5%)
	75+	11 (0.4%)	26 (1.0%)	16 (0.6%)	21 (0.8%)	37 (1.4%)
Gender	Female	707 (27.0%)	1009 (38.5%)	756 (28.9%)	960 (36.7%)	1716 (65.5%)
	Male	589 (22.5%)	314 (12.0%)	419 (16.0%)	484 (18.5%)	903 (34.5%)
Marital status	Single	664 (25.4%)	547 (20.9%)	518 (19.8%)	693 (26.5%)	1211 (46.2%)
	Married	605 (23.1%)	598 (22.8%)	580 (22.1%)	623 (23.8%)	1203 (45.9%)
	Others	27 (1.0%)	178 (6.8%)	77 (2.9%)	128 (4.9%)	205 (7.8%)
Highest level of education	High School Diploma	127 (4.8%)	92 (3.5%)	77 (2.9%)	142 (5.4%)	219 (8.4%)
	Technical Diploma	31 (1.2%)	84 (3.2%)	51 (1.9%)	64 (2.4%)	115 (4.4%)
	Bachelors (BA/BS)	401 (15.3%)	469 (17.9%)	378 (14.4%)	492 (18.8%)	870 (33.2%)
	Masters (MA, MSc)	515 (19.7%)	495 (18.9%)	483 (18.4%)	527 (20.1%)	1010 (38.6%)
	PhD and Post-Doctoral	86 (3.3%)	103 (3.9%)	93 (3.6%)	96 (3.7%)	189 (7.2%)
	Others	136 (5.2%)	80 (3.1%)	93 (3.6%)	123 (4.7%)	216 (8.2%)
Education/training in yoga science	Short-term course	132 (5.0%)	182 (6.9%)	160 (6.1%)	154 (5.9%)	314 (12%)
	Certificate in yoga	166 (6.3%)	241 (9.2%)	185 (7.1%)	222 (8.5%)	407 (15.5%)
	Diploma course	89 (3.4%)	336 (12.8%)	151 (5.8%)	274 (10.5%)	425 (16.2%)
	Bachelor’s degree in yoga	259 (9.9%)	61 (2.3%)	111 (4.2%)	209 (8.0%)	320 (12.2%)
	Master’s in yoga	287 (11.0%)	116 (4.4%)	205 (7.8%)	198 (7.6%)	403 (15.4%)
	PhD and post-doctoral	60 (2.3%)	35 (1.3%)	47 (1.8%)	48 (1.8%)	95 (3.6%)
	None	303 (11.6%)	352 (13.4%)	316 (12.1%)	339 (12.9%)	655 (25%)
Manage a yoga center/studio	Yes	553 (21.1%)	580 (22.1%)	536 (20.5%)	597 (22.8%)	1133 (43.3%)
	No	630 (24.1%)	646 (24.7%)	541 (20.7%)	735 (28.1%)	1276 (48.7%)
	In past	113 (4.3%)	97 (3.7%)	98 (3.7%)	112 (4.3%)	210 (8%)

**Table 2:** Subscale Correlation Matrix Correlation is significant at the 0.01 level (two-tailed). SS1: Acceptance/Compassion, SS2: Breathwork, SS3: Physicality, SS4: Active Postures, SS5: Restorative Postures, SS6: Body Locks (Bandhas), SS7: Body Awareness, SS8: Mental/Emotional Awareness, SS9: Health Benefits, SS10: Individual Attention, SS11: Social Aspects, SS12: Spirituality, SS13: Meditation/Mindfulness, SS14: Yoga Philosophy.

	SS1	SS2	SS3	SS4	SS5	SS6	SS7	SS8	SS9	SS10	SS11	SS12	SS13	SS14
SS1	1.000	0.614	0.404	0.427	0.510	0.402	0.532	0.601	0.530	0.330	0.287	0.443	0.511	0.468
SS2	0.614	1.000	0.468	0.520	0.564	0.473	0.603	0.575	0.519	0.322	0.226	0.402	0.464	0.382
SS3	0.404	0.468	1.000	0.631	0.444	0.498	0.462	0.424	0.424	0.459	0.433	0.342	0.335	0.347
SS4	0.427	0.520	0.631	1.000	0.619	0.531	0.621	0.467	0.384	0.431	0.293	0.321	0.334	0.286
SS5	0.510	0.564	0.444	0.619	1.000	0.556	0.646	0.606	0.476	0.402	0.329	0.383	0.437	0.355
SS6	0.402	0.473	0.498	0.531	0.556	1.000	0.557	0.506	0.427	0.413	0.367	0.394	0.423	0.409
SS7	0.532	0.603	0.462	0.621	0.646	0.557	1.000	0.683	0.516	0.396	0.277	0.370	0.471	0.370
SS8	0.601	0.575	0.424	0.467	0.606	0.506	0.683	1.000	0.683	0.406	0.377	0.494	0.632	0.531
SS9	0.530	0.519	0.424	0.384	0.476	0.427	0.516	0.683	1.000	0.521	0.463	0.637	0.678	0.679
SS10	0.330	0.322	0.459	0.431	0.402	0.413	0.396	0.406	0.521	1.000	0.622	0.519	0.487	0.517
SS11	0.287	0.226	0.433	0.293	0.329	0.367	0.277	0.377	0.463	0.622	1.000	0.572	0.503	0.536
SS12	0.443	0.402	0.342	0.321	0.383	0.394	0.370	0.494	0.637	0.519	0.572	1.000	0.729	0.754
SS13	0.511	0.464	0.335	0.334	0.437	0.423	0.471	0.632	0.678	0.487	0.503	0.729	1.000	0.787
SS14	0.468	0.382	0.347	0.286	0.355	0.409	0.370	0.531	0.679	0.517	0.536	0.754	0.787	1.000

Comparison of yoga practice across the four factors

Table 3 presents the mean scores for all 14 subscales across the four factors: region, sex, delivery mode, and participation status. The total EPYQ score for India (3.55) was higher than that for the United States (3.33). The remote delivery mode scored 3.45, while in-person delivery scored 3.42. The score for women (3.43) was marginally lower than that for men (3.45). Instructors had a higher score (3.52) compared to practitioners (3.33). The mean scores for each subscale across these factors highlight that social aspects (2.63) and individual attention (3.10) received the lowest scores, while breathwork (4.06) and body awareness (3.81) had the highest scores.

**Table 3:** Descriptive Statistics SS1: Acceptance/Compassion, SS2: Breathwork, SS3: Physicality, SS4: Active Postures, SS5: Restorative Postures, SS6: Body Locks (Bandhas), SS7: Body Awareness, SS8: Mental/Emotional Awareness, SS9: Health Benefits, SS10: Individual Attention, SS11: Social Aspects, SS12: Spirituality, SS13: Meditation/Mindfulness, SS14: Yoga Philosophy.

		SS1	SS2	SS3	SS4	SS5	SS6	SS7	SS8	SS9	SS10	SS11	SS12	SS13	SS14	Total
India	Mean	3.68	4.08	3.34	3.47	3.55	3.27	3.78	3.64	3.86	3.35	2.92	3.54	3.64	3.51	3.55
	SD	0.94	0.92	0.88	0.85	0.9	1.01	1.01	0.97	0.98	1.07	1.05	1.02	1.02	1.1	0.72
	Total	1296 (49.5%)	1296 (49.5%)	1296 (49.5%)	1296 (49.5%)	1296 (49.5%)	1296 (49.5%)	1296 (49.5%)	1296 (49.5%)	1296 (49.5%)	1296 (49.5%)	1296 (49.5%)	1296 (49.5%)	1296 (49.5%)	1296 (49.5%)	1296 (49.5%)
United States	Mean	3.72	4.04	3.05	3.49	3.55	3.19	3.84	3.61	3.49	2.85	2.34	3	3.4	3.02	3.33
	SD	0.92	0.85	0.9	0.83	0.84	1.04	0.91	0.93	1.04	1.16	1.03	1.15	1.06	1.17	0.69
	Total	1323 (50.5%)	1323 (50.5%)	1323 (50.5%)	1323 (50.5%)	1323 (50.5%)	1323 (50.5%)	1323 (50.5%)	1323 (50.5%)	1323 (50.5%)	1323 (50.5%)	1323 (50.5%)	1323 (50.5%)	1323 (50.5%)	1323 (50.5%)	1323 (50.5%)
Women	Mean	3.74	4.1	3.19	3.54	3.61	3.26	3.87	3.64	3.62	3.05	2.54	3.2	3.47	3.18	3.43
	SD	0.91	0.85	0.88	0.82	0.84	1.03	0.93	0.96	1.04	1.16	1.06	1.15	1.07	1.18	0.72
	Total	1716 (65.5%)	1716 (65.5%)	1716 (65.5%)	1716 (65.5%)	1716 (65.5%)	1716 (65.5%)	1716 (65.5%)	1716 (65.5%)	1716 (65.5%)	1716 (65.5%)	1716 (65.5%)	1716 (65.5%)	1716 (65.5%)	1716 (65.5%)	1716 (65.5%)
Men	Mean	3.62	3.98	3.19	3.38	3.44	3.18	3.68	3.59	3.78	3.19	2.79	3.4	3.61	3.42	3.45
	SD	0.94	0.94	0.93	0.87	0.91	1.03	1.01	0.94	0.99	1.1	1.08	1.06	1	1.12	0.72
	Total	903 (34.5%)	903 (34.5%)	903 (34.5%)	903 (34.5%)	903 (34.5%)	903 (34.5%)	903 (34.5%)	903 (34.5%)	903 (34.5%)	903 (34.5%)	903 (34.5%)	903 (34.5%)	903 (34.5%)	903 (34.5%)	903 (34.5%)
Instructor	Mean	3.81	4.13	3.17	3.47	3.65	3.25	3.93	3.74	3.8	3.17	2.68	3.38	3.66	3.42	3.52
	SD	0.9	0.89	0.9	0.85	0.87	1.05	0.95	0.94	0.97	1.12	1.06	1.1	1.02	1.11	0.7
	Total	1485 (56.7%)	1485 (56.7%)	1485 (56.7%)	1485 (56.7%)	1485 (56.7%)	1485 (56.7%)	1485 (56.7%)	1485 (56.7%)	1485 (56.7%)	1485 (56.7%)	1485 (56.7%)	1485 (56.7%)	1485 (56.7%)	1485 (56.7%)	1485 (56.7%)
Practitioner	Mean	3.55	3.97	3.22	3.49	3.41	3.2	3.64	3.47	3.51	3	2.56	3.13	3.34	3.05	3.33
	SD	0.93	0.87	0.9	0.82	0.86	1	0.95	0.94	1.07	1.16	1.1	1.14	1.06	1.2	0.73
	Total	1134 (43.3%)	1134 (43.3%)	1134 (43.3%)	1134 (43.3%)	1134 (43.3%)	1134 (43.3%)	1134 (43.3%)	1134 (43.3%)	1134 (43.3%)	1134 (43.3%)	1134 (43.3%)	1134 (43.3%)	1134 (43.3%)	1134 (43.3%)	1134 (43.3%)
Remote delivery mode	Mean	3.72	4.04	3.22	3.46	3.56	3.24	3.79	3.66	3.75	3.01	2.65	3.33	3.58	3.35	3.45
	SD	0.9	0.91	0.93	0.85	0.89	1.01	0.98	0.93	1.01	1.19	1.09	1.12	1.02	1.14	0.71
	Total	1175 (44.9%)	1175 (44.9%)	1175 (44.9%)	1175 (44.9%)	1175 (44.9%)	1175 (44.9%)	1175 (44.9%)	1175 (44.9%)	1175 (44.9%)	1175 (44.9%)	1175 (44.9%)	1175 (44.9%)	1175 (44.9%)	1175 (44.9%)	1175 (44.9%)
In-person delivery mode	Mean	3.68	4.07	3.17	3.5	3.54	3.22	3.82	3.6	3.62	3.18	2.61	3.22	3.46	3.19	3.42
	SD	0.94	0.86	0.87	0.83	0.86	1.04	0.95	0.97	1.04	1.09	1.06	1.13	1.07	1.18	0.72
	Total	1444 (55.1%)	1444 (55.1%)	1444 (55.1%)	1444 (55.1%)	1444 (55.1%)	1444 (55.1%)	1444 (55.1%)	1444 (55.1%)	1444 (55.1%)	1444 (55.1%)	1444 (55.1%)	1444 (55.1%)	1444 (55.1%)	1444 (55.1%)	1444 (55.1%)
Total	Mean	3.7	4.06	3.19	3.48	3.55	3.23	3.81	3.62	3.68	3.1	2.63	3.27	3.52	3.26	3.44
	SD	0.93	0.88	0.9	0.84	0.87	1.03	0.96	0.95	1.03	1.14	1.08	1.12	1.05	1.16	0.72
	Total	2619 (100%)	2619 (100%)	2619 (100%)	2619 (100%)	2619 (100%)	2619 (100%)	2619 (100%)	2619 (100%)	2619 (100%)	2619 (100%)	2619 (100%)	2619 (100%)	2619 (100%)	2619 (100%)	2619 (100%)

Comparison of yoga practice along the 14 components

Table 4 compares the mean differences for the 14 subscales by factor. In terms of region, the U.S. sample scored higher than the Indian sample on body awareness and acceptance/compassion (mean differences of -0.06 and -0.03, respectively). Conversely, the Indian sample scored higher on spirituality and social aspects (mean differences of 0.54 and 0.57, respectively). The higher mean score for spirituality in India may be due to yoga’s spiritual significance in the country, whereas yoga is often viewed as a mind/body practice in the West, as reflected in the U.S. sample’s higher scores for body awareness and acceptance/compassion.

**Table 4:** Mean Differences for the 14 Subscales by Factors SS1: Acceptance/Compassion, SS2: Breathwork, SS3: Physicality, SS4: Active Postures, SS5: Restorative Postures, SS6: Body Locks (Bandhas), SS7: Body Awareness, SS8: Mental/Emotional Awareness, SS9: Health Benefits, SS10: Individual Attention, SS11: Social Aspects, SS12: Spirituality, SS13: Meditation/Mindfulness, SS14: Yoga Philosophy.

Subscale	Mean	India, Mean	United States, Mean	Diff.	Women, Mean	Men, Mean	Diff.	Instructor, Mean	Practitioner, Mean	Diff.	Remote Delivery Mode, Mean	In-person Delivery Mode, Mean	Diff.
SS1	3.70	3.68	3.72	-0.03	3.74	3.62	0.12	3.81	3.55	0.26	3.72	3.68	0.03
SS2	4.06	4.08	4.04	0.05	4.10	3.98	0.12	4.13	3.97	0.15	4.04	4.07	-0.03
SS3	3.19	3.34	3.05	0.29	3.19	3.19	0.00	3.17	3.22	-0.05	3.22	3.17	0.05
SS4	3.48	3.47	3.49	-0.02	3.54	3.38	0.16	3.47	3.49	-0.02	3.46	3.50	-0.04
SS5	3.55	3.55	3.55	0.00	3.61	3.44	0.17	3.65	3.41	0.25	3.56	3.54	0.02
SS6	3.23	3.27	3.19	0.08	3.26	3.18	0.08	3.25	3.20	0.05	3.24	3.22	0.02
SS7	3.81	3.78	3.84	-0.06	3.87	3.68	0.19	3.93	3.64	0.29	3.79	3.82	-0.03
SS8	3.62	3.64	3.61	0.03	3.64	3.59	0.05	3.74	3.47	0.27	3.66	3.60	0.06
SS9	3.68	3.86	3.49	0.37	3.62	3.78	-0.16	3.80	3.51	0.28	3.75	3.62	0.13
SS10	3.10	3.35	2.85	0.50	3.05	3.19	-0.14	3.17	3.00	0.17	3.01	3.18	-0.17
SS11	2.63	2.92	2.34	0.57	2.54	2.79	-0.26	2.68	2.56	0.11	2.65	2.61	0.04
SS12	3.27	3.54	3.00	0.54	3.20	3.40	-0.20	3.38	3.13	0.25	3.33	3.22	0.11
SS13	3.52	3.64	3.40	0.23	3.47	3.61	-0.14	3.66	3.34	0.32	3.58	3.46	0.12
SS14	3.26	3.51	3.02	0.49	3.18	3.42	-0.24	3.42	3.05	0.37	3.35	3.19	0.17
Total	3.44	3.55	3.33	0.22	3.43	3.45	-0.02	3.52	3.33	0.19	3.45	3.42	0.03

Regarding delivery mode comparison (Table 4), remote yoga scored higher on the yoga philosophy and health benefits subscales (mean differences of 0.17 and 0.13, respectively) but lower on individual attention and active postures (mean differences of -0.17 and -0.04, respectively). These findings suggest that remote delivery effectively addresses both physical health and mental well-being. However, the mean differences between delivery modes were generally small for all subscales.

By sex (Table 4), men scored higher on social aspects and yoga philosophy (mean differences of -0.26 and -0.24, respectively), while women scored higher on body awareness and restorative postures (mean differences of 0.19 and 0.17, respectively). These results suggest that men may gain more social and philosophical benefits from yoga, while women focus more on physical awareness.

In terms of participation status, practitioners scored higher on physicality and active postures (mean differences of -0.05 and -0.02, respectively), whereas instructors scored higher on yoga philosophy and meditation/mindfulness (mean differences of 0.37 and 0.32, respectively). This indicates that as individuals transition from practitioners to instructors, their focus shifts from physical to mental and philosophical aspects of yoga.

As shown in Table 5, all subscales except for social aspects (2.63) had mean scores greater than 3, indicating effective delivery of yoga components. Table 6 presents the significant differences for the 14 subscales across the four factors, and Table 7 outlines the interaction effects. Significant differences were found by region and sex at the 5% level, with an interaction effect observed between region and participation status. Of the 14 subscales, health benefits, meditation/mindfulness, and yoga philosophy showed significant differences across all four factors. Finally, the test of between-participant effects (Table 8) revealed crucial differences in subscales and interaction effects, though the magnitude of these differences was notably low, warranting careful interpretation.

**Table 5:** Descriptive Statistics for the 14 Subscales

Subscales	N	Mean	Median	SD	% of Positive Responses (>3)
SS1 Acceptance/Compassion	2,619	3.6997	3.8000	0.9255	71.40%
SS2 Breathwork	2,619	4.0584	4.2000	0.8825	81.14%
SS3 Physicality	2,619	3.1899	3.1250	0.8986	53.26%
SS4 Active Postures	2,619	3.4800	3.5000	0.8389	66.40%
SS5 Restorative Postures	2,619	3.5486	3.6000	0.8715	68.38%
SS6 Body Locks (Bandhas)	2,619	3.2323	3.0000	1.0283	48.07%
SS7 Body Awareness	2,619	3.8069	4.0000	0.9644	71.36%
SS8 Mental/Emotional Awareness	2,619	3.6244	3.6000	0.9507	68.31%
SS9 Health Benefits	2,619	3.6756	3.7500	1.0260	68.08%
SS10 Individual Attention	2,619	3.1000	3.0000	1.1427	45.70%
SS11 Social Aspects	2,619	2.6264	2.3333	1.0764	29.67%
SS12 Spirituality	2,619	3.2680	3.2500	1.1225	53.23%
SS13 Meditation/Mindfulness	2,619	3.5177	3.5000	1.0482	63.54%
SS14 Yoga Philosophy	2,619	3.2621	3.2500	1.1632	52.96%

**Table 6:** Summary of the Significant Differences From the Multivariate Analysis

	Region		Sex		Delivery mode		Participation status	
Subscale	Diff.	P	Diff.	P	Diff.	P	Diff.	p
SS1 Acceptance/Compassion	-0.032	0.865	0.118	0.009	0.261	0.310	0.034	0.000
SS2 Breathwork	0.045	0.064	0.117	0.002	0.155	0.224	-0.026	0.000
SS3 Physicality	0.291	0.000	-0.001	0.124	-0.054	0.751	0.051	0.304
SS4 Active Postures	-0.023	0.238	0.160	0.000	-0.024	0.128	-0.039	0.204
SS5 Restorative Postures	0.003	0.030	0.166	0.000	0.245	0.600	0.015	0.000
SS6 Body Locks (Bandhas)	0.080	0.007	0.078	0.023	0.051	0.975	0.021	0.348
SS7 Body Awareness	-0.061	0.879	0.188	0.000	0.291	0.401	-0.031	0.000
SS8 Mental/Emotional Awareness	0.034	0.187	0.046	0.191	0.271	0.447	0.056	0.000
SS9 Health Benefits	0.371	0.000	-0.157	0.021	0.284	0.042	0.128	0.000
SS10 Individual Attention	0.498	0.000	-0.144	0.292	0.172	0.000	-0.172	0.000
SS11 Social Aspects	0.575	0.000	-0.256	0.000	0.114	0.976	0.038	0.006
SS12 Spirituality	0.544	0.000	-0.200	0.017	0.252	0.176	0.114	0.000
SS13 Meditation/Mindfulness	0.234	0.000	-0.143	0.005	0.318	0.019	0.120	0.000
SS14 Yoga Philosophy	0.495	0.000	-0.243	0.000	0.368	0.006	0.168	0.000

**Table 7:** Results of the Multivariate Analysis Hypothesis df =14, Error df = 2590

Effect	Value	F	p
Intercept	0.958	4183.376	<0.0000
Region	0.113	23.620	<0.0000
Sex	0.037	7.008	<0.0000
Delivery mode	0.027	5.052	<0.0000
Participation status	0.066	13.049	<0.0000
Region × Sex	0.017	3.227	<0.0000
Region × Delivery mode	0.014	2.633	<0.0008
Region × Participation status	0.014	2.584	<0.0010
Sex × Delivery mode	0.008	1.493	<0.1052
Sex × Participation status	0.010	1.836	<0.0288
Delivery mode × Participation status	0.006	1.085	<0.3661
Region × Sex × Delivery mode	0.007	1.360	<0.1642
Region × Sex × Participation status	0.007	1.261	<0.2237
Region × Delivery mode × Participation status	0.010	1.827	<0.0299
Sex × Delivery mode × Participation status	0.006	1.029	<0.4208
Region × Sex × Delivery mode × Participation status	0.011	1.967	<0.0168

**Table 8:** Tests of the Between-Participant Effects

Factor		Type III Sum of Squares	df	Mean Square	F	P
Region	SS1 Acceptance/Compassion	0.024	1	0.024	0.029	0.865
	SS2 Breathwork	2.645	1	2.645	3.434	0.064
	SS3 Physicality	45.811	1	45.811	58.155	0.000
	SS4 Active Postures	0.972	1	0.972	1.394	0.238
	SS5 Restorative Postures	3.469	1	3.469	4.709	0.030
	SS6 Body Locks (Bandhas)	7.610	1	7.610	7.215	0.007
	SS7 Body Awareness	0.021	1	0.021	0.023	0.879
	SS8 Mental/Emotional Awareness	1.541	1	1.541	1.743	0.187
	SS9 Health Benefits	54.743	1	54.743	55.071	0.000
	SS10 Individual Attention	134.414	1	134.414	111.303	0.000
	SS11 Social Aspects	139.254	1	139.254	130.703	0.000
	SS12 Spirituality	140.737	1	140.737	121.208	0.000
	SS13 Meditation/Mindfulness	22.583	1	22.583	21.460	0.000
	SS14 Yoga Philosophy	107.588	1	107.588	86.609	0.000
Sex	SS1 Acceptance/Compassion	5.671	1	5.671	6.771	0.009
	SS2 Breathwork	7.689	1	7.689	9.981	0.002
	SS3 Physicality	1.866	1	1.866	2.369	0.124
	SS4 Active Postures	14.851	1	14.851	21.290	0.000
	SS5 Restorative Postures	17.139	1	17.139	23.262	0.000
	SS6 Body Locks (Bandhas)	5.418	1	5.418	5.137	0.023
	SS7 Body Awareness	16.524	1	16.524	18.275	0.000
	SS8 Mental/Emotional Awareness	1.509	1	1.509	1.707	0.191
	SS9 Health Benefits	5.276	1	5.276	5.308	0.021
	SS10 Individual Attention	1.344	1	1.344	1.113	0.292
	SS11 Social Aspects	13.969	1	13.969	13.111	0.000
	SS12 Spirituality	6.635	1	6.635	5.714	0.017
	SS13 Meditation/Mindfulness	8.351	1	8.351	7.936	0.005
	SS14 Yoga Philosophy	15.822	1	15.822	12.736	0.000
Delivery mode	SS1 Acceptance/Compassion	0.863	1	0.863	1.031	0.310
	SS2 Breathwork	1.139	1	1.139	1.479	0.224
	SS3 Physicality	0.079	1	0.079	0.101	0.751
	SS4 Active Postures	1.621	1	1.621	2.323	0.128
	SS5 Restorative Postures	0.203	1	0.203	0.275	0.600
	SS6 Body Locks (Bandhas)	0.001	1	0.001	0.001	0.975
	SS7 Body Awareness	0.637	1	0.637	0.705	0.401
	SS8 Mental/Emotional Awareness	0.512	1	0.512	0.579	0.447
	SS9 Health Benefits	4.106	1	4.106	4.131	0.042
	SS10 Individual Attention	22.509	1	22.509	18.639	0.000
	SS11 Social Aspects	0.001	1	0.001	0.001	0.976
	SS12 Spirituality	2.123	1	2.123	1.828	0.176
	SS13 Meditation/Mindfulness	5.763	1	5.763	5.476	0.019
	SS14 Yoga Philosophy	9.258	1	9.258	7.453	0.006
Participation status	SS1 Acceptance/Compassion	30.331	1	30.331	36.213	0.000
	SS2 Breathwork	10.587	1	10.587	13.744	0.000
	SS3 Physicality	0.834	1	0.834	1.058	0.304
	SS4 Active Postures	1.126	1	1.126	1.614	0.204
	SS5 Restorative Postures	23.426	1	23.426	31.795	0.000
	SS6 Body Locks (Bandhas)	0.931	1	0.931	0.882	0.348
	SS7 Body Awareness	44.315	1	44.315	49.009	0.000
	SS8 Mental/Emotional Awareness	36.977	1	36.977	41.820	0.000
	SS9 Health Benefits	46.233	1	46.233	46.511	0.000
	SS10 Individual Attention	16.605	1	16.605	13.750	0.000
	SS11 Social Aspects	8.192	1	8.192	7.689	0.006
	SS12 Spirituality	31.782	1	31.782	27.371	0.000
	SS13 Meditation/Mindfulness	49.977	1	49.977	47.490	0.000
	SS14 Yoga Philosophy	74.181	1	74.181	59.716	0.000
Region × Sex	SS1 Acceptance/Compassion	0.682	1	0.682	0.814	0.367
	SS2 Breathwork	1.690	1	1.690	2.194	0.139
	SS3 Physicality	0.497	1	0.497	0.631	0.427
	SS4 Active Postures	1.555	1	1.555	2.229	0.136
	SS5 Restorative Postures	4.666	1	4.666	6.333	0.012
	SS6 Body Locks (Bandhas)	1.115	1	1.115	1.057	0.304
	SS7 Body Awareness	0.031	1	0.031	0.035	0.853
	SS8 Mental/Emotional Awareness	0.013	1	0.013	0.015	0.904
	SS9 Health Benefits	5.996	1	5.996	6.032	0.014
	SS10 Individual Attention	3.192	1	3.192	2.643	0.104
	SS11 Social Aspects	9.415	1	9.415	8.837	0.003
	SS12 Spirituality	4.565	1	4.565	3.931	0.048
	SS13 Meditation/Mindfulness	2.993	1	2.993	2.844	0.092
	SS14 Yoga Philosophy	4.427	1	4.427	3.564	0.059

## Discussion

This study aimed to identify the key factors that differentiate yoga practices, with the goal of improving the validity and comparability of scientific research and mitigating gaps in the integration of yoga into global healthcare. We conducted a cross-sectional analysis of 2,619 participants from India and the United States, examining differences based on four factors: region, sex, delivery mode, and participation status. Data were collected using the 14 subscales (properties) of the EPYQ, and the mean scores for each factor were analyzed.

This study’s cross-sectional analysis revealed several important insights. First, to avoid drawing broad conclusions, the study demonstrates the need for a more in-depth examination by identifying and analyzing each property (14 subscales or components) in yoga interventions. The study's approach aligns with the importance of analyzing yoga interventions by individual components or properties, as recommended by [25]. This approach will be critical for identifying key factors for validity and comparability.

Mean scores from Table 4 show that practices in both regions scored high on breathwork (4.06), body awareness (3.81), acceptance/compassion (3.71), and health benefits (3.68). However, components with lower scores included social aspects (2.63), individual attention (3.10), and body locks (bandhas) (3.23). The mean scores for each factor and subscale provide a baseline for yoga delivery in specific settings and populations. Analyzing these scores could help identify areas for improvement, enhance yoga delivery, and provide a systematic approach to improving these components.

Comparing geographic regions, the Indian population scored higher on the subscales for social aspects, spirituality, yoga philosophy, and individual attention. Meanwhile, the U.S. sample scored higher on body awareness and postures, reflecting the influence of Hatha yoga in the West. Overall, the total mean score for the Indian population (3.55) was higher than that for the United States (3.33), with significant differences across the various subscales.

The comparison of mean scores across all four factors demonstrates how different yoga practices exist around the world. This supports what other studies have found about the importance of taking into account the target population's background, culture, traditions, and beliefs when designing yoga programs [27].

Simultaneously, this study reinforces the caution of adopting a universal methodology for yoga delivery, emphasizing the need for a data-driven approach that considers the variability in yoga components across different populations. Other studies [22] have concluded that when designing studies and developing interventions, we must place greater methodological rigor on the selection of yoga intervention delivery methods.

Our findings align with previous research that examined the impact of individual properties (subscales), such as individualized attention [28], on the experiences and outcomes of yoga interventions, regardless of in-person or remote delivery. Other factors, such as tailored instruction, instructor support, and personalized feedback, are also critical for positive outcomes. These findings support the feasibility and effectiveness of online yoga interventions, as participants reported high levels of satisfaction, and the interventions were well-attended with strong practice rates [29].

We recommend further analysis of the subscale scores for yoga delivery modes to align with the specific goals of each intervention, thereby enhancing participant outcomes. These insights can be applied to improve the interface design and interaction methodologies of online yoga platforms.

This study had several limitations. It focused only on four factors of yoga delivery and did not explore other important factors, such as participants’ cultural beliefs, barriers to practice, adverse effects, or preferences. Furthermore, the study only included participants from India and the United States, thereby limiting the generalizability of the findings to other regions.

## Conclusions

Successfully integrating yoga into mainstream healthcare requires addressing the inherent variance in its practice. Our factor-based analysis identified significant differences across region, sex, delivery mode, and participation status. A post-pandemic study demonstrated that remote yoga has been widely accepted and proven comparable to in-person delivery in both Eastern and Western populations. The findings emphasize the need to customize yoga practices to address global diversity, enhancing both delivery and outcomes. This study, therefore, calls for replacing “black box” evaluations of yoga interventions, those lacking detailed measures and misidentifying key components, with a clear understanding of the macro- and micro-level factors influencing yoga practice. Researchers, yoga stakeholders, healthcare providers, and wellness practitioners can utilize these insights to create systematic yoga interventions that align with their objectives. Future research should further explore how region, sex, and participation status influence the effectiveness of remote yoga, contributing to improving the validity and reliability of yoga interventions.
